# Soft Tissue Reconstruction and Integration to Implant After Bone-Tumor Resection: A Current Concept Review

**DOI:** 10.3390/curroncol31110531

**Published:** 2024-11-15

**Authors:** Elisa Pesare, Raffaele Vitiello, Tommaso Greco, Giuseppe Solarino, Giulio Maccauro, Antonio Ziranu

**Affiliations:** 1 Orthopaedics Unit, Policlinico Universitario di Bari, Department of Translational Biomedicine and Neuroscience ‘DiBraiN’, University of Bari “Aldo Moro”, Piazza G. Cesare 11, 70124 Bari, Italy; e.pesare3@studenti.uniba.it (E.P.); giuseppe.solarino@uniba.it (G.S.); 2 Department of Orthopaedics, Fondazione Policlinico Universitario A. Gemelli IRCCS, Università Cattolica del Sacro Cuore, 00168 Rome, Italy; giulio.maccauro@policlinicogemelli.it (G.M.); antonio.ziranu@unicatt.it (A.Z.); 3 Dipartimento di Scienze della Vita, della Salute e delle Professioni Sanitarie, Link Campus University, 00165 Rome, Italy; tommaso.greco2@guest.policlinicogemelli.it

**Keywords:** soft tissue, integration, reconstruction, megaprosthesis, soft tissue coverage

## Abstract

Introduction: With the advancements in chemotherapy for malignant bone tumors, the number of patients eligible for limb salvage surgery has increased. Surgeons face a subsequent challenge in limb-sparing resection due to the need for reconstructing soft tissue coverage. The aim of this review is to focus on the present state of the field in these areas, highlighting recent advancements. Methods: A literature research was conducted using keywords such as “soft tissue”, “integration”, “reconstruction”, “megaprosthesis”, and “soft tissue coverage”, on different databases, and following the Preferred Reporting Items for Systematic Reviews and Meta-analyses (PRISMA) criteria, a total of 35 studies were selected. Results: In recent times, there has been a growing emphasis on different techniques such mesh application, allograft-prosthesis composites, allograft reconstruction, a polyethylene terephthalate (PET) tube, prosthesis itself and certain metals utilized for implant coatings are used in soft tissue reconstruction. Conclusion: While tissue-engineered constructs and advancements in biological and cellular approaches have shown potential for enhancing osseointegration and interactions with soft tissues and implants, the actual clinical outcomes have frequently fallen short of expectations. The success of soft tissue integration is crucial for achieving functional outcomes, minimizing complications, and ensuring the long-term stability of orthopedic implants.

## 1. Introduction

Various multimodal treatment strategies based on tumor histology, combining different therapies are used depending on the type of tumor. However, tumor excision with a wide margin is the gold standard surgical treatment in each case.

With the advent of adjuvant and neoadjuvant chemotherapy and advancements in pre- or post-operative systemic therapies for malignant bone tumors, the number of patients eligible for limb salvage surgery has increased. Their mortality rates have been proven to be comparable to those patients managed with amputation [1,2,3].

A surgical option for skeletal reconstruction after extensive bone resections due to malignant tumors of long bones involves the implantation of megaprostheses, offering high mechanical resistance properties.

When properly selected, today reconstruction with modular or custom-made megaprostheses results in stronger and more stable construction than in the past.

Technology and techniques, along with materials such as titanium or cobalt-chrome, have improved, providing long-lasting, biocompatible options that support bone ingrowth into the prosthesis.

The wide resection of the tumor results in a large segmental defect: regeneration is needed in a quantity far beyond the normal potential of self-healing of patients.

In addition to prosthetic replacement, there are other different procedures such as autogenous grafts, arthrodesis and composite allografts, which have all been described in literature [4] but if the muscle anchorage is not adequate, the limb salvage procedure loses its potential benefits.

Furthermore, the reconstruction of soft tissue around megaprostheses is challenging because it is often associated with functional deficits resulting from the removal of tendons and their attachments, and in this case, the prosthesis behaves just like a bone spacer without preserving the stable and mobile articular functionality [4]: preserving limb function is one of the goals of the surgical treatment.

Probably instead of replacing tissues with inert medical devices, a better choice should be more biological approaches that focus on the repair and reconstruction of tissue structure and function but currently, there are limited procedures available for soft tissue attachment to implants and to satisfy the need for long-term repair [5].

Tissue engineering have been utilized to improve the interaction between soft tissues and implants [6] but the integration of tissues on the surface depends on many variables such as materials used and the tension of the construct.

Providing adequate soft tissue coverage is crucial in postoperative management to avoid complications such as wound problems and early secondary infections [4]. Despite favorable long-term implant longevity in recent decades, the main causes of revision surgery remain aseptic or septic loosening, material malfunction, dislocation, migration, and soft tissue failures with poor implant covering [7,8].

Therefore, in orthopedic oncology, the interface between soft tissues and implanted prostheses and grafts has become an area of significant interest. The aim of this review is to focus on the present state of the field in these areas, highlighting recent advancements and outlining potential future directions.

## 2. Materials and Methods

The Preferred Reporting Items for Systematic Reviews and Meta-analyses (PRISMA) [9] guidelines were employed to conduct a literature search.

The research was conducted using keywords such as “soft tissue”, “integration”, “reconstruction”, “megaprosthesis”, and “soft tissue coverage”, along with Boolean operators AND and OR.

Two members (E.P. and R.V.) collected the literature, by searching in several databases (PubMed, Medline, Cochrane, and Google Scholar) generating a total of 586 articles.

Titles were examined by two authors independently (E.P. and A.Z.). Two second researchers (T.G. and G.S.) verified articles, identified and collected some data on a Microsoft Excel spreadsheet like the first author, title, design of the study, and year of publication.

**Figure 1:**
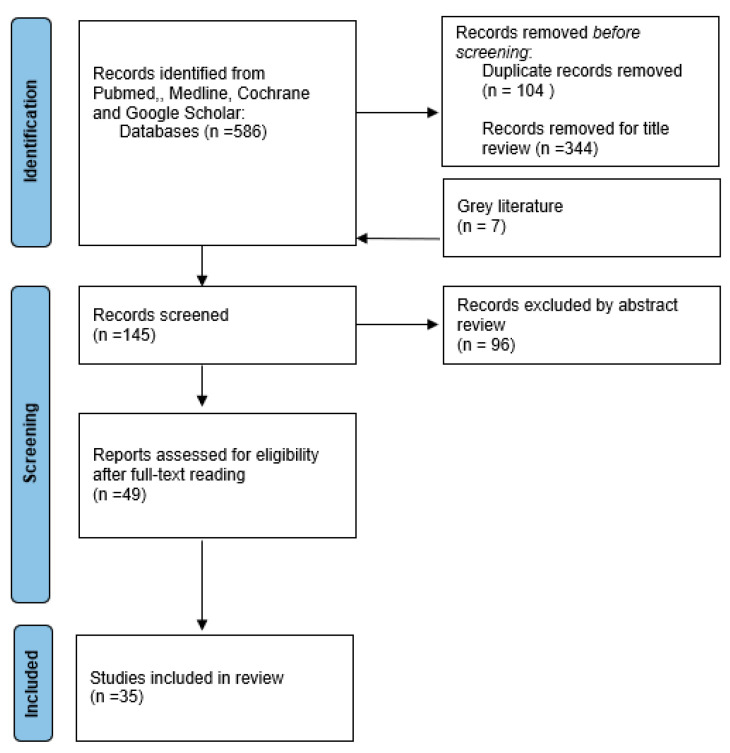
PRISMA flow chart.

The gray literature was examined to identify additional missed research by exploring extensively each published study’s bibliography and identifying pertinent items that may have been missed. The screening process is shown in the study review progression flowchart (Figure 1).

English-language articles were selected, deleting other languages papers, and duplicates were removed.

In the beginning, we focused on including reviews, systematic reviews, and meta-analyses, all of which provide a high level of evidence. However, we later decided to broaden our research by examining clinical trials, in vivo studies, technical notes, and expert opinions, aiming for a much more comprehensive selection.

This expansion likely broadened the range of data sources we considered, allowing us to gather a more diverse set of insights and perspectives on the research. By including clinical trials and in vivo studies, we aim to have more direct evidence from experimental studies, while we thought technical notes and expert opinions could provide valuable practical insights and expert perspectives.

Lots of articles didn’t fit the topic and were excluded by titles, and then by abstract review. In the following phase, authors independently examined papers selected by full-text reading and in case of disagreement on studied inclusion a very experienced surgeon on tumor removal was approached to settle the dispute (G.M.). In the end, thirty-five published studies were included after more than 551 papers were removed (see Table 1).

## 3. Results

Soft-tissue reconstruction plays a crucial role in providing coverage for the prosthesis, significantly reducing the incidence of wound problems and early secondary infections [3]. Various procedures, such as prosthetic reconstruction, allograft-prosthesis composites (APC), and allograft reconstruction, have been documented in the literature [1].

In an ideal scenario [6], the reconstruction of large bony defects should not only restore anatomy but also optimize function while minimizing the risk of implant failure and the need for revision. Restoring limbs function at the same time as maintaining overall survival rates and reducing local tumor recurrence is critical for sustaining the quality of life of these patients. While autografts remain a viable option for smaller defects, their use for reconstructing larger segments is limited due to associated donor-site morbidity.

Recent research efforts have been directed toward optimizing the interactions of bone and soft tissue with metallic implants and osseous grafts, aiming to prevent infection on implanted materials.

The development of implant coatings has benefited greatly from the application of tissue engineering ideas. The purpose of these coatings is to lower the risk of infection and improve osseointegration. Likewise, these methods have been applied to enhance the interaction between implants and soft tissues [6].

Additionally, there is a growing emphasis on expanding reconstructive options by incorporating tissue-engineered grafts.

Below, we will list some of the most commonly used procedures with some examples:

### 3.1. Mesh Augmentation with Biological Enhancements

In arthroplasty and oncology different biological enhancements are frequently employed in conjunction with mesh to improve tendon integration with the implant surface.

According to Sundar et al. [10] mesh with demineralized bone matrix (DBM) has been studied in ovine models as augmentation of the tendon attachment to a metallic implant.

DBM is a processed form of bone that has had its mineral content removed, by decalcification of cortical bone, leaving behind the organic matrix and growth factors [15].

DBM is rich in glycoproteins and other bioactive molecules that can stimulate bone formation and aid in the healing process. It is less immunogenic than an allograft bone graft.

They show stem cell populations present in healing interfaces could be responsible for the formation of bone and cartilage in the neo-entheses. DBM augmentation has a positive effect on early tendon-bone implant healing with good functional weight-bearing [10].

Even Higuera et al. [11], hypothesized that the reconstitution of the direct tendon-bone insertion morphology in tendon reattachment to a metallic implant could be achieved using an allogenic cancellous bone plate augmented with autogenous cancellous bone and marrow, and that the autogenous bone grafts could be replaced by recombinant human osteogenic protein-1 (rhOP-1). However, their results indicate that the incorporation of the bone allograft as a transitional structure between the tendon and the metallic surface needs improvement, as they showed a bony ingrowth of less than 1% over their implant surface in that tendon-implant model.

Pendegrass et al. [12] studied how augmentation of the tendon-implant interface with a bone block could improve the retention of the graft on the implant surface. They demonstrated that graft retention is improved by using an autologous bone block and marrow graft to augment the healing tendon-implant interface. Their study showed that bone block augmentation of tendon-implant interfaces results in a return to pre-operative levels of weight-bearing by 24 weeks and in a superior functional outcome.

There are some studies describing mesh augments as a reliable choice to improve patients’ functional outcomes.

### 3.2. Biological Reconstruction

Biological reattachment of tendons to metallic prostheses is not directly possible and often results in a loss of muscle strength. The Allograft Prosthesis Composites (APC) were developed to address the challenges associated with megaprostheses and overcome this limitation [16,37]. APC is born as a surgical technique that combines a prosthesis with allografts: it consists of a revision-type prosthesis cemented onto a skeletal allograft, allowing the residual soft tissue sleeve to be biologically fixed around it [17].

The aim is to leverage the advantages of prostheses while achieving functional improvement through the biological reattachment of tendons to muscles, ultimately enhancing stability [6].

Allografts offer several advantages over metal implants due to their resemblance to the host’s biology [18]. Additionally, osteoarticular grafts, including allograft reconstruction, facilitate the anatomical reconstruction of joints. This approach helps maintain specific anatomical sites for tendon and soft tissue attachments, contributing to improved stability and overall function. The most crucial factor during allograft selection is ensuring a shape match [19,20].

Reconstruction success percentages are highly dependent on the reconstruction location; 82% to 84% of reconstructions succeed in this regard [21].

However, this method is not without complications. Common complications associated with this reconstruction procedure include periprosthetic bone resorption, rejection reactions, allograft bone fracture, infection, non-union, and the risk of disease transmission [17]. Approximately fifty-four percent of patients will require surgery again due to these complications [21]. Muscolo et al. [18] reported a survival rate of 81% at 10 years for composite prostheses.

According to several studies, allograft fracture and deep infection are frequently cited as the primary reasons for revision in Allograft Prosthesis Composites (APC) [22]. Moreover, infection rates in APC have been reported to range from 0% to 24% [22].

Indeed, another potential risk of failure associated with these methods is the recipient’s immune system rejecting the allograft. Allograft rejection is an immunological response to the transplanted tissue, as the immune system perceives the allograft as foreign, leading to a reaction against the transplanted material [20].

Advances in bone preservation and processing today have reduced inaccuracies in fitting the graft to the patient, as highlighted in recent developments [6].

Results of APCs revision rate have been reported to be 29% and that value changes in relation to the anatomic sites: proximal humerus APCs showed the lowest revision rate [23].

For example, Ruggieri et al. [24] reported a revision rate of 14.2% after performing a series of proximal humerus reconstructions utilizing Allograft Prosthesis Composites (APC). Similarly, in proximal humerus deformities treated with APC, Abdeen et al. [16] reported an 8.3% revision rate.

According to recent clinical research, there is a greater frequency of problems (estimated at 60%) when osteoarticular allografts are used in the proximal tibia or distal femur, instead [21].

Additionally, it seems that both the revision and infection rates are higher with irradiated allografts than with fresh-frozen ones [23].

### 3.3. Implant Coatings Metals

**Figure 2:**
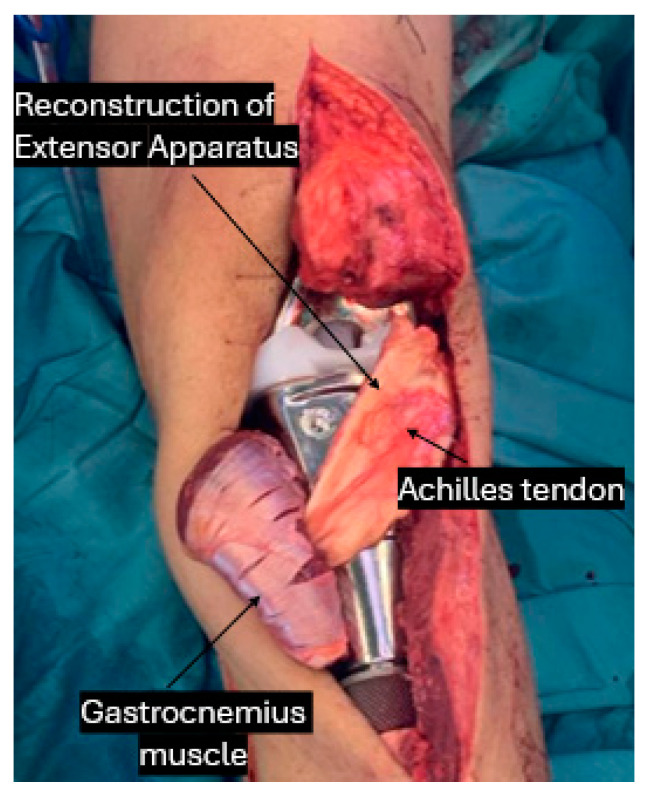
Proximal tibial osteosarcoma with en bloc resection and reconstruction of extensor apparatus on the implant surface.

Moreover, it has recently been established that certain metals used for implant coatings, primarily due to their physical properties, enhance the recruitment and adherence of mesenchymal cells while promoting osteogenesis through biological processes [38]. Porous metals are being suggested as a therapeutic approach for promoting healing in tendon-to-implant and tendon-to-bone connections (Figure 2) in press-fit fixated implants.

Furthermore, due to its biomechanical qualities, materials such as porous tantalum are becoming more and more popular for tumor prosthesis surgery [39]. Several studies have compared the mechanical and biological characteristics of tantalum with those of titanium, and tantalum appears to be frequently employed, particularly because of its remarkable toughness, corrosion resistance, and bioactivity [39]. Tantalum is less reactive than titanium due to a smaller range of Young’s modulus, according to research done by Fan et colleagues [39].

Additionally, further data suggest its superior osteogenic differentiation capabilities compared to titanium [25]. Porous tantalum structures show exceptional strength, which makes them very useful for rebuilding significant bone lesions. This material demonstrates the ability to enhance the compression and bearing strength as osseous integration progresses, typically occurring around 4–6 weeks after surgery [25]. Bone ingrowth into porous tantalum implants is widely documented in the literature, but there are also sporadic reports of soft tissue extending and integrating into this material. This phenomenon described is associated with stronger long-term osteoid development and increased soft tissue stability, indicating the potential for longer durability of the implant over time free of complications [25]. According to these investigations previously mentioned, porous tantalum facilitates reintegration with the implant [25]: it seems to have the ability to encourage cell adhesion and proliferation.

Notably, the use of porous tantalum at attachment sites, such as the supraspinatus and patellar tendons, has already shown nearly physiological strength.

In an in vitro study [26], tantalum-coated glass caused a significant increase in the proliferation of human fibroblasts after 14 days of culture, with no quantifiable negative effects observed on fibroblast and human mesenchymal stem cell behavior.

According to S. Reach [27], when the initial interface mechanical environment is carefully controlled, a highly porous form of tantalum metal would allow the ingrowth of tendon tissue with clinically relevant tendon-to-implant fixation strength.

Soft tissue ingrowth into porous tantalum was noted also in a murine model of rotator cuff repair at the site of supraspinatus insertion [28], supporting previous reports and researches mentioned.

Tucker et al. [28]. evaluated the effectiveness of P2 porous titanium-coated implants in promoting supraspinatus tendon-to-bone healing in acute supraspinatus detachment and repair using a rat model. Their results showed substantial tissue ingrowth at all postoperative time periods and better mechanical properties in the P2 implant group at 2 and 4 weeks after surgery as compared to traditional supraspinatus repair.

Of particular note is the significant increase in maximum load observed in the P2 implant group, with a 76% increase at 2 weeks and a 41% increase at 4 weeks compared to the standard repair group. These results suggest the potential of P2 porous titanium-coated implants in augmenting and enhancing the outcomes of supraspinatus tendon-to-bone repair.

Certainly, the study has several limitations that warrant consideration. Firstly, it utilized an experimental animal model, which may not fully reflect the complexities of human anatomy and physiology. Specifically, the small size of the rat rotator cuff imposes constraints on the applicability of similar repair techniques used in clinical practice.

Additionally, the study focused on acute supraspinatus detachment and repair, whereas many supraspinatus tears encountered in clinical settings are often subacute or chronic. This discrepancy in the timing of injury and repair may impact the generalizability of the study findings to real-world scenarios.

These findings collectively support the notion of porous metals (such as tantalum) having favorable properties for enhancing outcomes in implant surgeries, especially in tumor prosthesis procedures. Therefore, porous tantalum could be an appropriate biomaterial to use in situations where soft tissue requires direct reattachment to implants and may also stimulate soft tissue healing.

### 3.4. Synthetic Meshes and Tubes

The mesh can be made from both synthetic and biological materials and is shaped to fit the specific defect. It is fixed with various techniques to the remaining healthy bone.

Ichikawa et al. [13] described the utilization of a synthetic mesh for extensor reconstruction after proximal tibial resection, attaching it to the tibial component of the prosthesis and describing it as a successful method.

Similarly, it evaluates a microporous biomaterial, a polypropylene (PPP) mesh to reconstruct the shoulder joint capsule and promote soft tissue reattachment after tumor resection [14]. Patients with humerus resection often face joint instability due to non-functional rotator cuff tendons and capsule. They support the use of nonabsorbable PPP mesh to reduce the rate of glenohumeral joint instability and dislocation, thus improving the patient’s quality of life.

Over a decade of in vivo and preclinical data on the biological enhancement of soft-tissue attachments to bone and metal have shown promise, but effective translation to clinical applications is still limited and requires further exploration.

Other biomaterial products that have gained recent popularity include polytetrafluoroethylene (ePTFE) and polyethylene terephthalate (PTT) [14], which are utilized for repairing functional soft tissues. Due to their very small pore diameters, these biomaterials may inhibit the ingrowth of surrounding soft tissues and the subsequent integration of biomaterials into soft tissues.

With positive clinical outcomes, the polyethylene terephthalate (PET) tube is employed to promote soft tissue and joint capsule reattachment surrounding the implant [1].

The practice of suturing soft tissues over a PET tube surrounding a megaprosthesis as a stable anchoring method following excision for bone malignant neoplasms is supported by published literature [29]. Additionally, this approach typically produces satisfactory functional outcomes in the majority of cases.

In cases of extraarticular resection, this tube is connected to the residual bone or the remaining part of the capsular structures. This tube allows the reattachment of the remaining muscles and tendons to the tube, providing the ingrowth of fibroblasts which enables the development of a stable joint-situation [30,31]. All attachments to the PET tube are made with non-absorbable sutures. It is characterized by a 200 m porous structure and a tensile strength of 4000 N.

PET tubes can be used on the gastrocnemius muscle for patients with proximal tibia endoprosthesis [32,33]. It can also be used to assist in the rebuilding of the rotator cuff in patients with a prosthetic proximal humerus [34].

For instance, Olsson et al. [1] described functional results in 20 patients treated with proximal humerus replacement using the PET tube. Their findings indicated that the use of the PET tube helped to prevent flail shoulder and dislocation.

In proximal femoral replacement, refixation of the iliopsoas muscle [32] and the gluteal muscles is performed through the PET tube. It not only allows the reattachment of the extensor apparatus, muscular structures, and remaining soft tissue in general [35], but also offers joint stability and permits early mobilization of patients [1].

**Figure 3:**
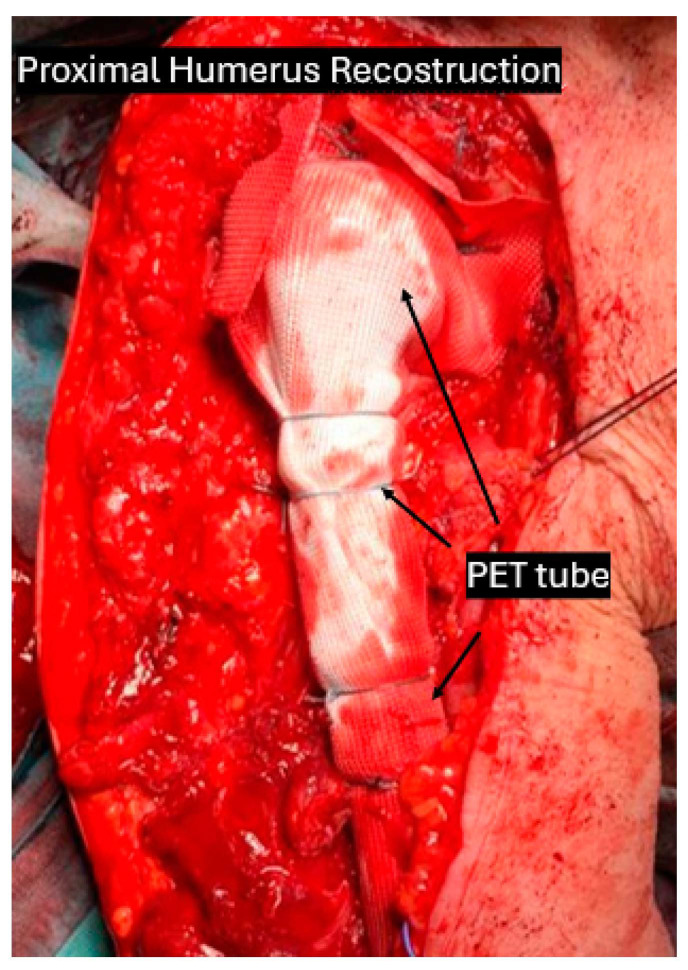
A case of chondrosarcoma of the proximal humerus which underwent total humerus resection and reconstruction with latissimus dorsi flap and PET tube.

Given the adherence of soft tissues to the device, the use of a PET-coated modular or custom-made megaprosthesis could be recommended for early functional rehabilitation. This makes the device particularly beneficial for young, active patients aiming to restore optimal musculoskeletal function as quickly as possible following these devastating and invasive procedure [29] (Figure 3).

Anyway, the use of the PET tube is not uniform in literature and represents an “open question” [29].

There are some studies analyzing the eventual correlation between using postoperative infections and the use of PET. Although most patients with malignant tumors have a higher infection risk due to chemotherapy and/or radiotherapy connected immunosuppression status, in most cases it seems PET tube was not associated with a statistically significant higher rate of infection [31] even among these immunosuppressed patients.

### 3.5. Prosthesis

Prosthesis has become an important means and development trend for limb salvage reconstruction, when compared with allograft, because it permits early mobilization, weight bearing [36] and mitigation of the risk of disease transmission from donor tissue [7,40].

The problem is that this reconstruction does not restore bone stock or provide anatomic locations for soft tissue attachments [2,6]. Most of the time, to ensure soft tissue around the implant, muscles are tensioned and sutured through the holes of the prosthesis [4].

Channels are fabricated on the tendon attachment point of the surface of the metal prosthesis to facilitate the suturing of tendons into the channels. In this manner, the tendon and bone are initially connected by sutures during the early stage of repair, and later on, they become integrated through cicatricial tissue formation [40].

Fixation of the tendon to the prosthesis may modify the direction forces of tension and change mobility of joints and function of muscle to which the tendon is connected.

Therefore, fixation of the tendon to the surface of the prosthesis is recommended and is beneficial for transmitting the tension to the prosthesis and reducing the occurrence of joint dislocation. Dislocation rates ranged from 1.7% and 11.1% (for hemiarthroplasty and 6.5% and 22% for total hip replacement) [29].

There are some studies in which patients with attachment of intact soft tissue by single sutures directly on the implant didn’t dislocate [32].

Another way is fixing tendons to the normal bone or in other parts through tendon transposition with changes in the function of such tendons. This method has been shown to achieve only 15% of the original strength, when the tendon is normally attached to the bone [40]. This leads to weakened functional characteristics of the tendon.

**Figure 4:**
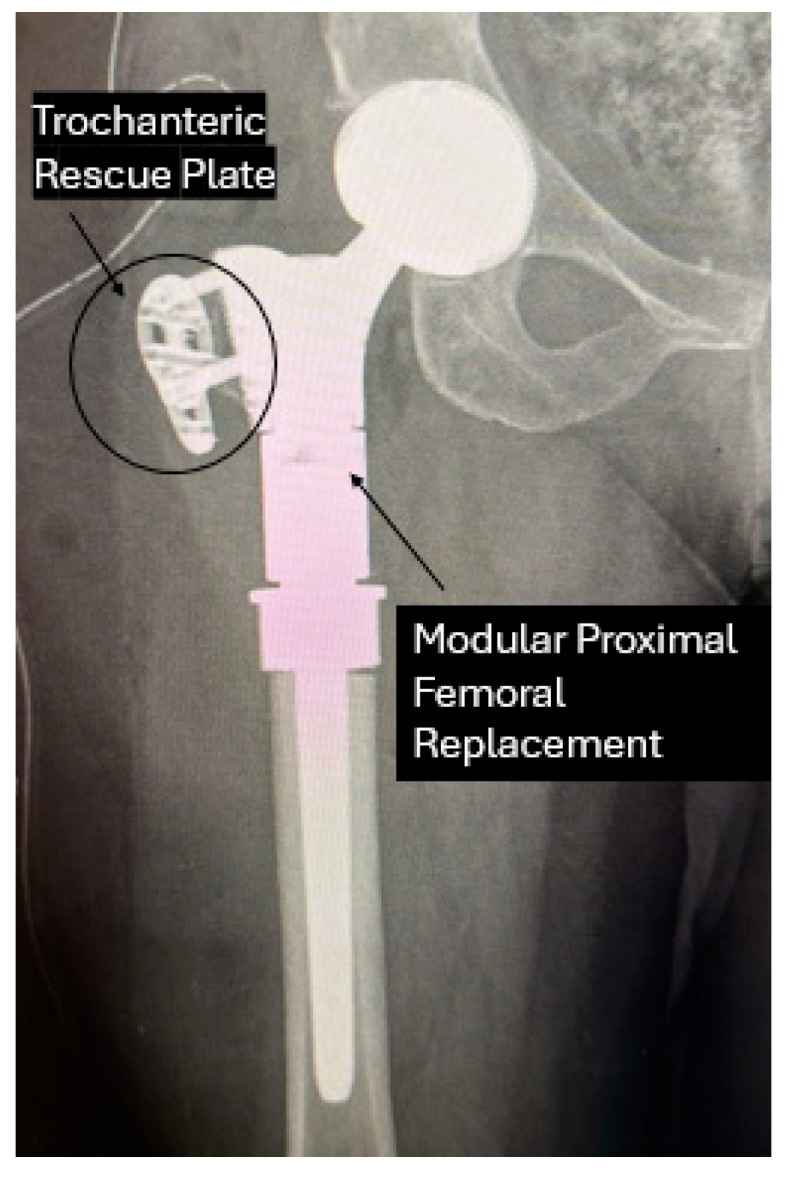
Modular proximal femoral replacement with trochanteric rescue plate for soft tissue fixation.

Furthermore, certain implants with modular components can retain muscle attachment sites. It is possible to save the trochanteric area for instance by using a prosthesis with a modular soft tissue fixation plate (Figure 4).

Although it is difficult to spare bone stock in oncology due to the necessity to be radical and avoid recurrence, it aids in the preservation of a favorable postoperative result.

Nowadays, research is focused on enhancing the interaction at the bone-metal contact in order to increase stability and avoid infection [40].

After limb-salvage surgery, there is a long-term risk of local infection, with a postoperative infection incidence of 8% to 15% [21]. This risk is increased by neoadjuvant chemotherapy, substantial resection, and long-segment tumor metal prosthesis placement [21].

Specifically, for lower limb tumor endoprostheses, the infection rate ranges from 8% to 10% [21]. The majority of these infections occur within 2 years post-operation, with staphylococcal infections being the most common type [21]. However, nearly 70% of deep infections arise within 12 months post-operation [21].

Once infected, the amputation rate can vary widely, ranging from 23.5% to 87% [21].

These data underscore the critical importance of close monitoring and implementing preventative measures in limb-salvage surgery to minimize the occurrence of postoperative infections.

## 4. Discussion

In our experience with limb resection procedures, we have found that utilizing a PET (polyethylene terephthalate) tube significantly enhances the stability of the implant and improves functional outcomes, thereby reducing the risk of dislocation. Importantly, we have observed that this method does not appear to increase the risk of infection, which is a crucial consideration in surgical practices.

On the other hand, the adoption of trochanteric rescue plates presents challenges, primarily due to the longer learning curve associated with their use, as noted by our team of experts. Prosthesis does not restore anatomical integrity of soft tissue attachments, leading to potential joint instability and infection risks. This complexity makes the integration of such techniques less straightforward in clinical settings compared to the PET tube method.

Mesh Augmentation with Biological Enhancements stands out for its ability to improve tendon integration with metallic implants, but its clinical translation remains limited, necessitating further research to optimize application. Similarly, Allograft-Prosthesis Composites (APC) leverage the strengths of allografts and prosthetic devices to achieve successful biological reattachment of tendons.

Incorporating implant coatings with Metals has the potential for fostering soft tissue ingrowth, but the limited long-term data and variability in surgical techniques suggest caution in widespread implementation. The difference between each technique is well summarized in Table 2.

It is also important to note that the procedures we typically perform are based on the extensive experience of a single, skilled surgeon. The sites for implant placement are varied and often unique, adding another layer of complexity to our analysis. Furthermore, advancements in 3D printing technology have opened new avenues in limb salvage surgery.

However, we believe that the choice of surgical strategy must always be tailored to the individual patient’s needs. A comprehensive preoperative plan is essential in determining the most effective approach, especially given the high complexity inherent in these procedures. Orthopedic surgeons must have the confidence to proceed with methods they are most comfortable with, ensuring they can adequately meet the needs of their patients. In cases where a surgeon feels less skilled in a particular procedure, seeking assistance from a more experienced colleague is not just advisable; it is imperative for ensuring optimal patient care and outcomes. This collaborative approach can bridge gaps in expertise and ultimately enhance the quality of care provided to patients undergoing limb salvage surgery.

## 5. Conclusions

We discussed the most common strategies currently used to address the reconstruction of soft tissue coverage in limb salvage surgery while preserving limb function and joint mobility. We described implant coatings that promote soft tissue ingrowth, allograft-prosthesis composites (APC), attachment sites for direct soft tissue integration onto the prosthesis, and the use of polyethylene terephthalate tube and mesh augments.

Despite the availability of various choices, determining the most effective strategy remains difficult for achieving the greatest postoperative results.

As a matter of fact, while tissue-engineered constructs and advancements in biological and cellular approaches have shown potential for enhancing osseointegration and interactions with soft tissues and implants, the actual clinical outcomes have frequently fallen short of expectations. Even explorations into innovative materials and devices have yielded disappointing results when applied in practical settings.

Consequently, there is a pressing need for new studies to address these challenges and seek improvements in the effectiveness of these technologies.

The success of soft tissue integration is crucial for achieving functional outcomes, minimizing complications, and ensuring the long-term stability of orthopedic implants.

## Figures and Tables

**Table 1:** Study selection characteristics.

Authors	Title	Type of Study	Year
**Mesh augmentation with biological enhancements**			
**Sundar et al.** [10]	Tendon re-attachment to metal prostheses in an in vivo animal model using demineralised bone matrix.	In vivo animal model	2009
**Higuera et al.** [11]	Tendon reattachment to a metallic implant using an allogenic bone plate augmented with rhOP-1 vs. autogenous cancellous bone and marrow in a canine model.	In vivo animal model	2005
**Pendegrass et al.** [12]	A comparison of augmentation techniques for reconstruction of the extensor mechanism following proximal tibial replacement in an experimental animal	In vivo animal model	2008
**Ichikawa et al.** [13]	A new technique using mesh for extensor reconstruction after proximal tibial resection	Retrospective study	2015
**Wang et al.** [14]	Endoprosthetic reconstruction of the proximal humerus after tumour resection with polypropylene mesh.	Retrospective study	2015
**Brink et al.** [15]	The choice between allograft or demineralized bone matrix is not unambiguous in trauma surgery.	Review	2021
**Allograft-prosthesis composites (APC)**			
**Abdeen et al.** [16]	Allograft-prosthesis composite reconstruction of the proximal part of the humerus. Functional outcome and survivorship.	Clinical trial	2009
**Gautam et al.** [17]	Megaprosthesis versus Allograft Prosthesis Composite for massive skeletal defects	Review	2018
**Muscolo DL et al.** [18]	Massive allograft use in orthopedic oncology.	Review	2006
**Cartiaux O et al.** [19]	Surgical inaccuracy of tumor resection and reconstruction within the pelvis An experimental study.	Experimental model	2008
**Paul L et al.** [20]	Selection of massive bone allografts using shape-matching 3-dimensional registration.	Experimental model	2010
**Xu M et al.** [21]	Guideline for Limb-Salvage Treatment of Osteosarcoma.	Review	2020
**Gautam et al.** [22]	Megaprosthesis Versus Allograft Prosthesis Composite for the Management of Massive Skeletal Defects: A Meta-Analysis of Comparative Studies.	Review	2021
**Aurégan et al.** [23]	Effect of anatomic site and irradiation on the rates of revision and infection of allograft-prosthesis composites after resection of a primary bone tumor: a meta-analysis.	Meta-Analysis	2016
**Ruggieri et al.** [24]	Preliminary results after reconstruction of bony defects of the proximal humerus with an allograft-resurfacing composite.	Retrospective study	2011
**Implant coatings metals**			
**Fan H. et al.** [25]	Highly Porous 3D Printed Tantalum Scaffolds Have Better Biomechanical and Microstructural Properties than Titanium Scaffolds.	Preclinical study	2021
**C. A. Gee et al.** [26]	The influence of tantalum on human cell lineages important for healing in soft-tissue reattachment surgery: an in-vitro analysis	In vitro- study	2019
**Reach et al.** [27]	Direct tendon attachment and healing to porous tantalum: An experimental animal study.	In vivo-animal model	2007
**Tucker et al.** [28]	P2 porous titanium implants improve tendon healing in an acute rat supraspinatus repair model.	In vivo-animal model	2017
**Polyethylene terephthalate (PTT) tube**			
**Wang et al.** [14]	Endoprosthetic reconstruction of the proximal humerus after tumour resection with polypropylene mesh.	Retrospective study	2015
**Gosheger et al.** [1]	Soft tissue reconstruction of megaprostheses using a trevira tube	Clinical trial	2001
**Oliva et al.** [29]	Hip megaprosthesis in oncological surgery: open questions.	Review	2019
**Sambri et al.** [30]	Silver-coated (PorAg^®^) endoprosthesis can be protective against reinfection in the treatment of tumor prostheses infection.	Retrospective study	2020
**Schmolders et al.** [31]	Silver-coated endoprosthetic replacement of the proximal humerus in case of tumour—is there an increased risk of periprosthetic infection by using a trevira tube?	Clinical trial	2017
**Bischel et al.** [32]	En-bloc resection of metastases of the proximal femur and reconstruction by modular arthroplasty is not only justified in patients with a curative treatment option—an observational study of a consecutive series of 45 patients.	Retrospective study	2020
**Puetzler et al.** [33]	Hip transposition procedure due to osteosarcoma metastasis of the ilium in a patient with preexisting rotationplasty leads to satisfactory functional result: A case report.	A case report	2020
**El Motassime et al.** [34]	Functional Outcomes and Shoulder Instability in Reconstruction of Proximal Humerus Metastases.	Retrospective study	2023
**Apostolopoulos et al.** [35]	Total elbow replacement for giant-cell tumor of bone after denosumab treatment: a case report.	A case report	2023
**Prosthesis**			
**Ham et al.** [36]	Limb salvage surgery for primary bone sarcoma of the lower extremities: Long-term consequences of endoprosthetic reconstructions.	Retrospective study	1998
**Hao-Ran et al.** [7]	Application and Development of Megaprostheses in Limb Salvage for Bone Tumors Around the Knee Joint.	Review	2022
**Burke et al.** [6]	Reconstructive Science in Orthopedic Oncology.	Review	2018
**Manfrini et al.** [2]	Evolution of surgical treatment for sarcomas of proximal humerus in children: Retrospective review at a single institute over 30 years.	Retrospective study	2018
**Sirveaux et al.** [4]	Reconstruction techniques after proximal humerus tumour resection	Review	2019
**Oliva et al.** [29]	Hip megaprosthesis in oncological surgery: open questions.	Review	2019
**Bischel et al.** [32]	En-bloc resection of metastases of the proximal femur and reconstruction by modular arthroplasty is not only justified in patients with a curative treatment option—an observational study of a consecutive series of 45 patients.	Retrospective study	2020
**Puetzler et al.** [33]	Hip transposition procedure due to osteosarcoma metastasis of the ilium in a patient with preexisting rotationplasty leads to satisfactory functional result: A case report.	A case report	2020

**Table 2:** The structured table summarizes the advantages and disadvantages of each technique discussed, according to the study selected.

**Mesh augmentation with biological enhancements**	**Advantages**	− Increase early tendon-bone healing − Utilizes synthetic and biological materials shaped for specific defects. − Can enhance graft retention on implant surfaces.
	**Disadvantages**	− Requires further exploration and research for optimization. − Potential for insufficient bony ingrowth in some models.
**Allograft-prosthesis composites (APC)**	**Advantages**	− Resemblance to the host’s biology − Facilitate the anatomical reconstruction of joints − Combination help biological reattachment of tendons.
	**Disadvantages**	− Reconstruction location variability of success rate − Shape matching dependent − Allograft rejection risk due to immune response.
**Implant coatings metals**	**Advantages**	− Documented success in bone and soft tissue ingrowth. − Enhance the compression and bearing strength − Encourage cell adhesion and proliferation
	**Disadvantages**	− Limited research on its long-term effectiveness − Variability due to differences in implantation technique.
**Polyethylene terephthalate (PTT) tube**	**Advantages**	− Stable anchoring method − Early functional rehabilitation − Promotes joint stability. − Demonstrates satisfactory functional outcomes in various reconstructions
	**Disadvantages**	− Potential inhibition of surrounding soft tissue ingrowth due to small pore sizes − Mixed literature on its effectiveness, requiring more standardization
**Prosthesis**	**Advantages**	− Early mobilization and weight-bearing − Lower risk of disease transmission associated with allografts − Flexible design options for integrating soft tissue fixation
	**Disadvantages**	− Reconstruction does not restore bone stock or provide anatomic locations for soft tissue attachments − Altered biomechanics may lead to joint instability or dislocation − Long-term infection risks (8–15% postoperative infection rates)

## Data Availability

Not applicable.
